# Enhanced Photocatalytic Activity of WS_2_ Film by Laser Drilling to Produce Porous WS_2_/WO_3_ Heterostructure

**DOI:** 10.1038/s41598-017-03254-2

**Published:** 2017-06-09

**Authors:** Sainan Ma, Longlui Zeng, Lili Tao, Chun Yin Tang, Huiyu Yuan, Hui Long, Ping Kwong Cheng, Yang Chai, Chuansheng Chen, Kin Hung Fung, Xuming Zhang, Shu Ping Lau, Yuen Hong Tsang

**Affiliations:** 1 Department of Applied Physics and Materials Research Center, The Hong Kong Polytechnic University, Hung Hom, Kowloon, Hong Kong People’s Republic of China; 2 The Hong Kong Polytechnic University Shenzhen Research Institute, Shenzhen, People’s Republic of China

## Abstract

Methods and mechanisms for improvement of photocatalytic activity, are important and popular research topics for renewable energy production and waste water treatment. Here, we demonstrate a facile laser drilling method for engineering well-aligned pore arrays on magnetron-sputtered WS_2_ nanofilms with increased active edge sites; the proposed method promotes partial oxidation to fabricate WS_2_/WO_3_ heterojunctions that enhance the separation of photogenerated electron-hole pairs. The WS_2_ film after one, two, and three treatments exhibited photocurrent density of 3.9, 6.2, and 8 μA/cm^2^, respectively, reaching up to 31 times larger than that of pristine WS_2_ film along with greatly improved charge recombination kinetics. The unprecedented combinational roles of laser drilling revealed in this study in regards to geometric tailoring, chemical transformation, and heterojunction positioning for WS_2-_based composite nanomaterials create a foundation for further enhancing the performance of other 2D transition metal dichalcogenides in photocatalysis via laser treatment.

## Introduction

Since the successful development of graphene, 2D layered materials have attracted tremendous research interests. The material is limited in terms of practical application, however, due to its zero-bandgap properties caused by valence and conduction bands meeting at the K points of the Brillouin zone^1^. In recent years, 2D layered transition metal dichalcogenides (TMDs) have become a popular research topic by virtue of their tunable bandgaps^2^, strong light-matter interactions^3^, extreme strain sustainability^4^, valley-selective optical stark effect^5^, and enhanced catalytic properties^6^. Tungsten disulfide (WS_2_), a typical layered TMD, consists of many layers of trigonal prismatic coordinated S-W-S planes connected to each other by van der Waals-type interactions. They offer novel functionalities over photodetectors^7^, lithium ion batteries^8^, solar cells^9^, optical limiters^10^, and hydrogen evolution reaction (HER) catalysts^11^. Compared to Pt-group metals, WS_2_ with modest Gibbs’ free energy is non-precious and earth-abundant, representing a remarkable potential alternative in electrochemical energy applications. The bandgap of semi-conductive WS_2_ is closely related to its layer numbers, ranging from 1.3 to 2.1 eV as it changes from bulk to fewer layers and exhibiting strong quantum confinement effect^12^, direct band gap transition^13^ and large spin-orbit coupling^14^, all of which make it a very promising photocatalyst.

Heterojunction design is an efficient strategy to extend photocurrent responses and facilitate rapid charge transfer via matched energy levels to inhibit electron-hole pair recombination^15^. Visible-light responding WO_3_/WS_2_ heterojunctions have been developed by hydrothermal/gas phase reaction^16^, in-situ anodic oxidation^17^, spray pyrolysis/sulfurization^18^, sputtering/plasma sulfurization^19^, and have exhibited both high optical absorption coefficients and catalytic activity. Compared against bulk WS_2_, the lateral heterostructure of WS_2_/WO_3_ is capable of much greater degradation of methyl orange and higher photocurrent response^20^. The slow decrease in photocurrent observed on the WS_2_/WO_3_ composite when the light was switched off reflects the favorable recombination properties of electron/hole pairs in the heterojunction^21^. Although several synthesis routes have been proposed to fabricate heterostructure, the effects of laser treatment on the formation of WS_2_/WO_3_ heterostructure remain unclear.

Thermodynamically, the formation of basal plane sites is more preferential than the formation of catalytically active edge sites^22^. The surface energy of the edge site was almost two orders of magnitude higher than that of the basal plane^23^. Structural design to expose active edge sites has been extensively investigated in recent years. Structures including highly ordered double-gyroid MoS_2_ bicontinous network^24^, defect-rich MoS_2_ ultrathin nanosheets^25^, and vertically aligned layered MoS_2_ films^26^, have been successfully developed to produce abundant active edge sites. Despite the notable progress made in the synthesis of two-dimensional metal chalcogenides, the geometric tailoring of porous WS_2_/WO_3_ heterostructure by laser treatment remains unexplored. Laser technique has been demonstrated as an attractive method to easily improve the photocatalytic properties of nanomaterials^27, 28^. The enhanced photocatalytic water splitting activity by laser treated TiO_2_ has been reported due to the structure modification under the laser irradiation^29^. Laser treatment of TMDs such as laser-thinning of bulk MoS_2_ into single layer and defect engineering of WSe_2_ films^30, 31^, represents a controllable strategy to increase material properties. In this study, magnetron sputtering and a facile, nanosecond pulse laser treatment was used to prepare porous WS_2_/WO_3_ heterojunctions to explore a new laser drilling technique for the geometric tailoring, chemical transformation, and heterojunction positioning of the material. The laser facilitated selective oxidation and increased number of active edge sites, in effect significantly enhancing the photoelectrocatalytic performance as well as the fast transportation of photogenerated electrons in the heterogeneous composite catalyst.

## Results and Discussion

**Figure 1:**
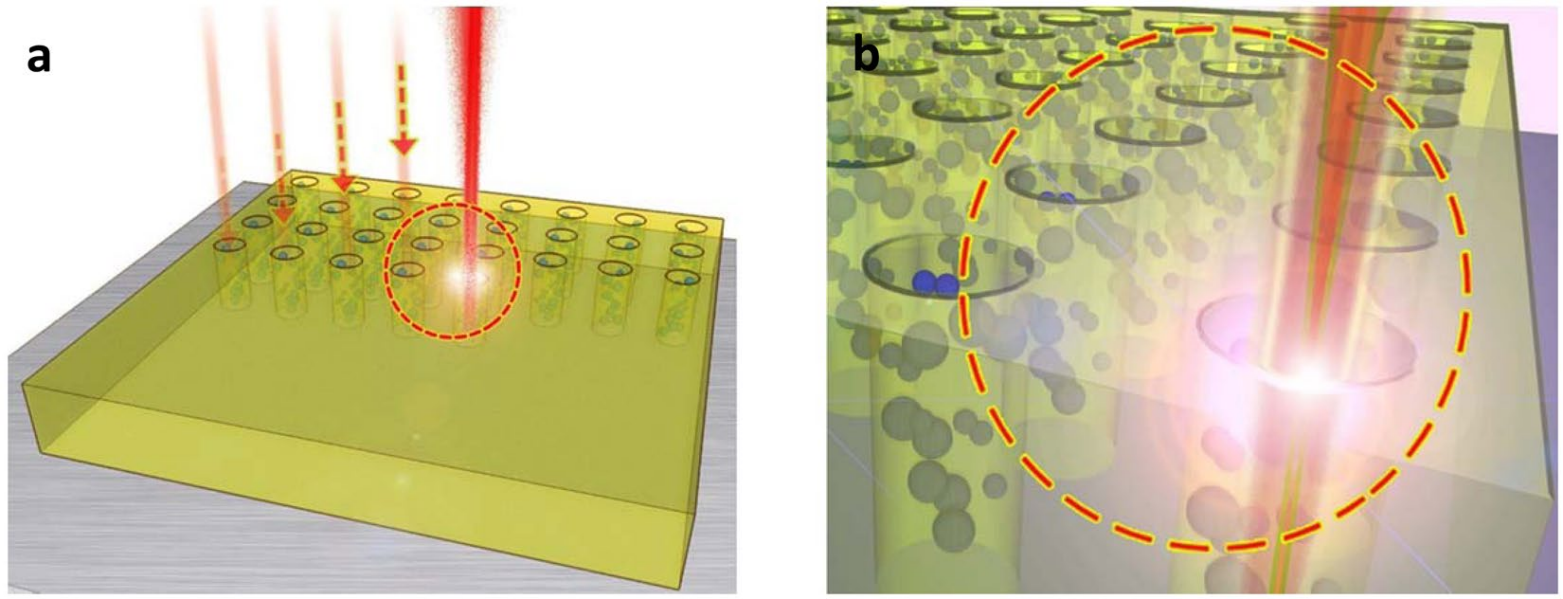
(**a**) Schematic diagram of nanosecond pulse laser treatment on samples with a moving laser beam. (**b**) Zoom-in structural model of porous WS_2_ film after laser treatment.

Laser materials processing is a popular technology employed in industry and the laser system used in this study is a low-cost widely and commercially available Q-switched Yb doped fiber laser with operational wavelength at near infrared, providing a cost effective way to modify materials for large area. Figure 1a shows a schematic diagram of the proposed laser treatment on the WS_2_ surface via a laser drilling setup with nanosecond pulse beam. When the laser radiation focuses on the surface of WS_2_, the laser heat flux, q_in_, at a distance ‘R’ from the center of the laser beam on the surface of the sample with Gaussian distribution as-expressed by Eqs (1) and (2)^32^. The consistent irradiation can result in the melting, ionization, bond breakage, gasification and phase explosion of the selected area.

1 $${q}_{in}=\frac{2\alpha {P}_{p}}{\pi {r}^{2}}exp(\frac{-2{R}^{2}}{{r}^{2}})$$

2 $$r=\frac{d}{2}[1+({M}^{2}\times \frac{4\lambda ({Z}_{m}+{f}_{c})}{\pi {d}^{2}})]$$

where *α* is the material absorption coefficient, *P*_*p*_ is the instantaneous laser power, *r* is the effective laser beam radius (which varies with the depth of hole), *δ*(*t*) is a pulse function, *d* is the laser beam diameter, *M* is the beam quality parameter, *λ* is to the laser wavelength, *Z*_*m*_ is the melt depth and *f*_*c*_ is the focal length of the focusing lens. These equations indicate that the heat flux on the materials surface is depending laser beam diameter, laser power and material absorption coefficient with respect to the input wavelength. Generally, the heat-affected zone and depth, which is characterized by structural changes of the laser irradiated sheet due to penetration of thermal wave, increases with the laser pulse energy delivery to the sheet. This proportional relationship allows for the control of the depth and affected zone of drilled WS_2_ film by altering the laser process parameters. Figure 1b shows the zoom-in structural model of orderly aligned, porous WS_2_ arrays after laser treatment containing many small particles around the holes. In order to form an applicable porous structure, the experimental parameters were selected as laser power of 0.92 W, λ = 1064 nm, pulse duration of 47 ns, repetition frequency of 50 kHz, scanning speed of ~1800 mm s^−1^ (See Supplementary Figs S1 and S2 for laser characterization details).

**Figure 2:**
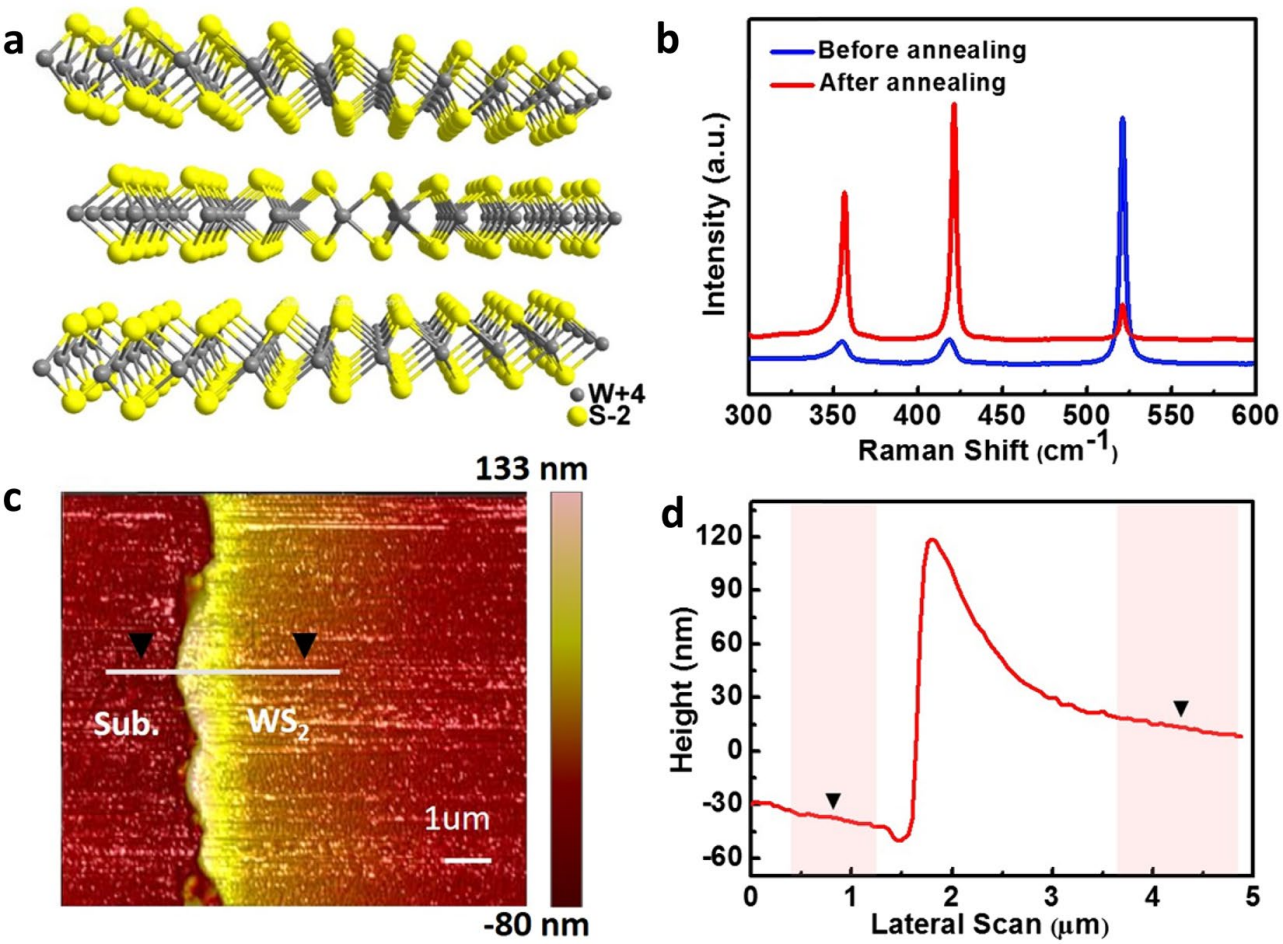
(**a**) Side view of the atomic structure of WS_2_ layers. (**b**) Raman spectra of WS_2_ films on Si substrate before and after annealing. (**c**) AFM image of annealed WS_2_ film with edge area. (**d**) Height information along the white line marked in (**c**).

Hexagonal (2H) and octahedral (1T) lattice structures are the main stable crystal phases of synthesized WS_2_ thin films. They facilitate controllable optical and electronic properties by allowing the designer to fine-tune the atomic distribution. Figure 2a shows the 2H semi-conductive phase of WS_2_ where strong, W-S covalent bonding within the single molecular layer combined with van der Waals interactions with a standard stack spacing of 0.618 nm create strong anisotropy. Figure 2b shows the Raman spectra of sputter-deposited WS_2_ film before and after annealing. The crystallinity of the treated WS_2_ film was greatly enhanced compared to the pristine sputtered film as revealed by a stronger peak intensity post-annealing at 800 °C. In addition to the peak at 521.0 cm^−1^ for the underlying substrate, two typical peaks at 356.7 cm^−1^ and 421.5 cm^−1^ appeared corresponding to the in-plane E^1^_2g_ and out-of-plane A_1g_ vibration from the WS_2_ crystalline film. The difference in shift excitation of the Raman spectra between E^1^_2g_ and A_1g_ modes (Δ = A_1g_ − E^1^_2g_) was 64.8 cm^−1^, indicating that the synthesized nanofilms consisted of several WS_2_ layers^33, 34^. Figure 2c shows the topography of WS_2_ film as-measured by AFM. We observed a cluster effect-dependent, small hill at the edge of the film where the coverage of WS_2_ was continuous. Generally, film thickness is dependent on sputtering parameters such as radio frequency power, gas pressure, and deposition time^35^; under the optimized conditions, the films were deposited at a rate of approximately 5 nm min^−1^. Figure 2d shows the corresponding height information along the white line marked in Fig. 2c, where the thickness of both sputtered and annealed WS_2_ films was ~51.8 nm.

**Figure 3:**
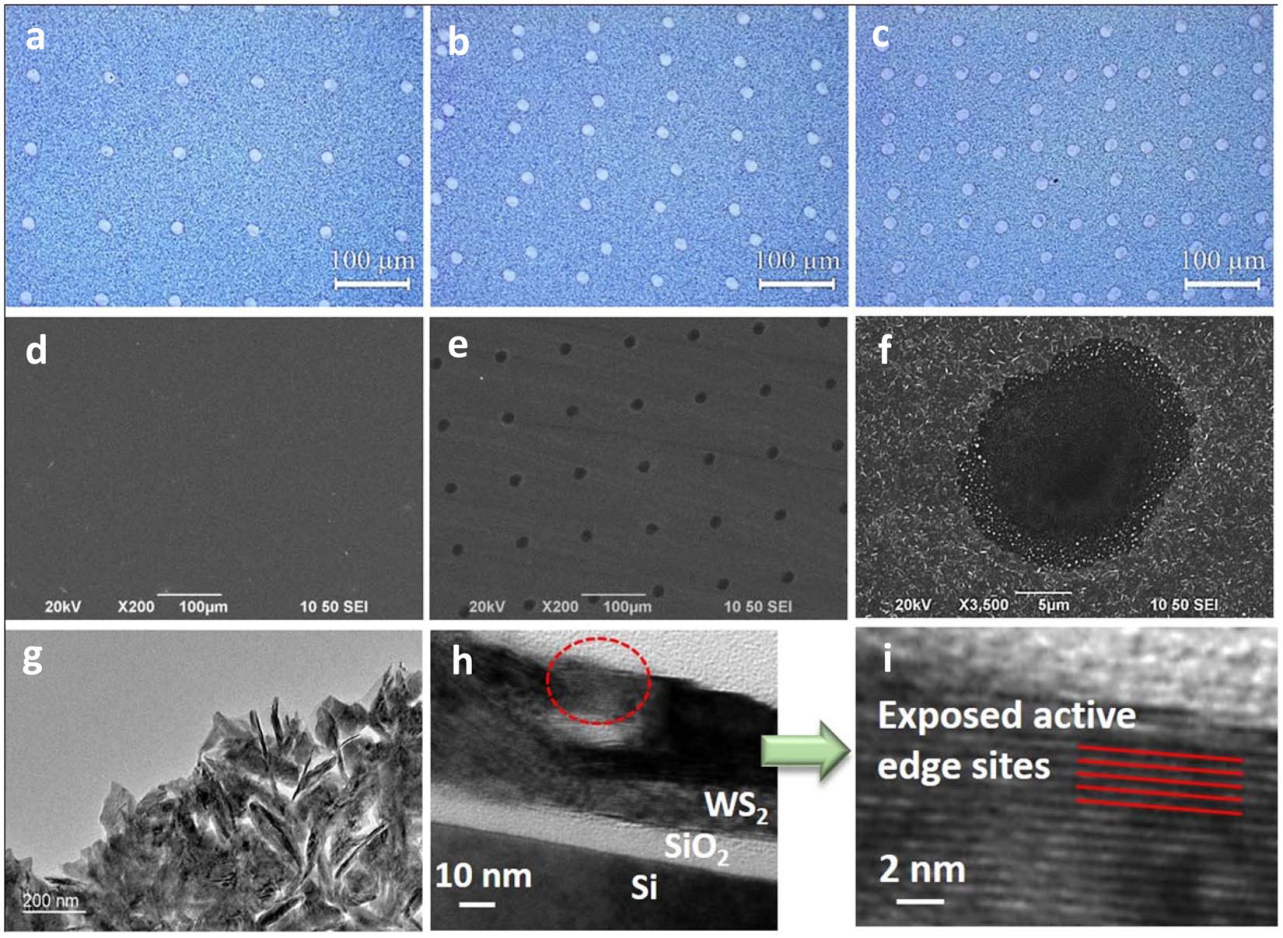
Optical images of WS_2_ film with laser treatment (**a**) once; (**b**) twice; (**c**) three times. SEM images of WS_2_ film (**d**) before laser treatment; (**e**,**f**) after one laser treatment. (**g**) TEM image of transferred WS_2_ film with laser-drilled hole. (**h**) HRTEM cross-section image of laser treated WS_2_ film. (**i**) Zoom-in HRTEM image of the hole area as indicated in (**h**) by red dot circular.

We exposed the same WS_2_ sample under successive laser treatment once, twice and three times; the resulting optical images are shown in Fig. 3a–c, respectively. Several microscale pores were in orderly alignment over the sample surface, and the pores density increased with the number of laser treatments. Figure 3d and e show SEM images of the WS_2_ film before and after laser drilling at low magnification, respectively. The WS_2_ film fabricated by sputtering and annealing was relatively smooth at low magnification. As shown in Fig. 3e, the average distance between the adjacent pores was ~80 μm. The average diameter of a single hole was ~17 μm, as shown in Fig. 3f. There was an abundance of small particles surrounding the hole, the smallest diameter of which reached ~62 nm. The unique formation of small particles observed was likely influenced by the fast transition from the overheated liquid to a mixture of vapor and drops during the vaporization of the laser-focused area^36^. The TEM image shown in Fig. 3g reveals that vertically oriented and horizontally aligned WS_2_ nanosheets coexisted in the samples. Further TEM characterization of the layers spacing within WS_2_ sample can be found in Supplementary Fig. S3. The layer-to-layer spacing was measured to be 0.63 nm (See Supplementary Fig. S3a), which is close to the standard 2H-WS_2_. Furthermore, based on horizontally aligned WS_2_ layers the interplanar spacing was 0.27 nm (See Supplementary Fig. S3b), which is consistent with the (100) plane of hexagonal WS_2_ nanosheets^37^. Figure 3h shows a HRTEM cross-section image of the laser treated WS_2_ film deposited on the silicon substrate. There was about ~10 nm silicon oxidation layer observed due to the natural oxidization. From the zoom-in HRTEM image shown in Fig. 3i, which is magnified picture of the part indicated by a red dot circle shown in Fig. 3h, the exposed active edge sites inside the hole can be clearly identified, which are highly catalytically active compared to the basal planes^38^. It is expected the overall photo-catalytic activity of this sample can be significantly enhanced by these exposed active edge sites.

**Figure 4:**
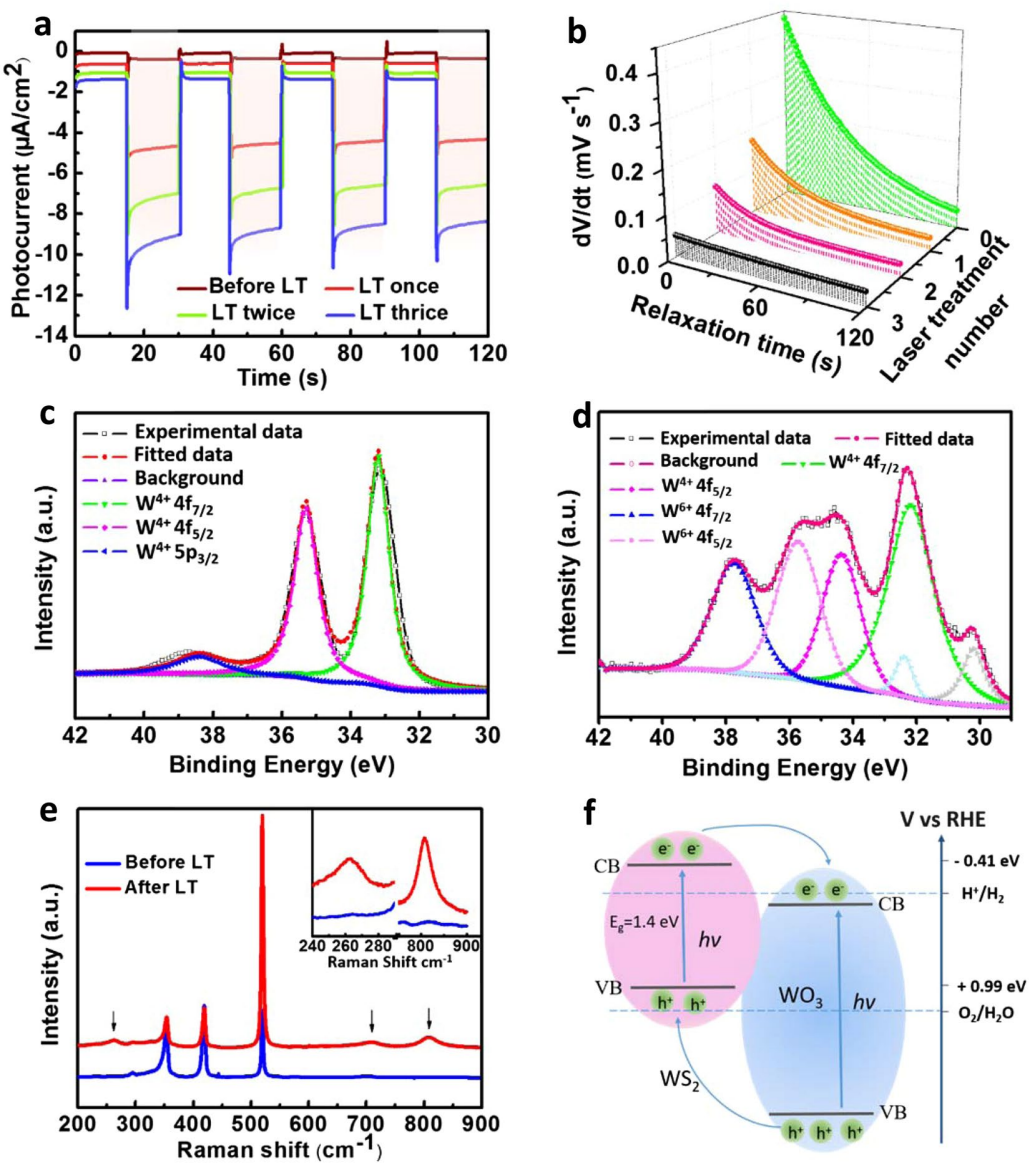
(**a**) Photocurrent as a function of time under solar simulated illumination (420 nm cut-off) without bias of the corresponding WS_2_ before and after one, two, and three laser treatments. (**b**) Electron lifetime measurements obtained based on decay of open circuit potential in a dark environment. XPS spectra of W 4f core level peak region for WS_2_ (**c**) before and (**d**) after laser treatment. (**e**) Raman spectra of WS_2_ before and after laser treatment. Inset shows two magnified peaks from 240 to 290 cm^−1^, and 750 to 900 cm^−1^. (**f**) Schematic diagram of band structure and charge transfer in WS_2_/WO_3_ heterostructure under visible light irradiation. (LT represents to laser treatment).

To further investigate the influence of laser treatment on photoelectrical activity of the samples, *I-t* measurements were taken to examine the photocurrent density of the WS_2_ film within 120 s light on/off cycles (Fig. 4a). The photocurrent densities of the silicon substrate with WS_2_ film was 0.25 μA cm^−2^ at 0 V versus Ag/AgCl. After the WS_2_ samples were subjected to laser treatment once, twice, and three times, the obtained photocurrent densities were 3.9, 6.2, and 8 μA cm^−2^, respectively. In other words, the photocurrent density of the sample laser-treated three times improved up to 31 times higher than that of the pristine sample. The photocurrent densities of pure silicon substrate were measured as reference, being 0.03 μA cm^−2^ at 0 V versus Ag/AgCl (See Supplementary Fig. S4), of which the photoelectrical activity is relatively poor. The photocurrent reproduced by these samples under light ON/OFF repeatedly is very consistent. There was a substantial current spike when the light was turned on due to the rapid recombination of excessive holes with electrons in quick succession^39^ after the separation of photoexcited electron-hole pairs. As the depth of laser drilled hole is about 156 nm under the average laser power of 0.92 W (See Supplementary Fig. S5), the hole has located on the substrate across the WS_2_ thin film. To exclude the influence of silicon substrate on the photocatalytic activity, the photocurrents of silicon wafer before and after laser treatment three times were conducted and compared with the performance of Si/WS_2_ after laser treatment three times. Relatively the photocurrent of silicon substrate with three times laser treatment is ignorable compared to silicon wafer with WS_2_ film deposition treated under the same conditions (See Supplementary Fig. S6 for details). Over the course of subsequent experiments, we found that the photocurrent density continually increased as the number of laser treatments increased; this further confirmed that laser drilling is an efficient way to enhance photocurrent. After repeating the laser scanning of nearly 20 times, the sample showed maximum photocurrent density achieved up to ~20 μA cm^−2^. Overlapping holes became more frequent as the treatment repetition number increased due to limitations in our laser setup, so the optimized value appeared within 15–25 treatments.

To better understand the improved photoelectrocatalytic performance observed, open-circuit photovoltage decay (OCPVD) was conducted to characterize the inherent electronic properties of the samples. OCPVD is an efficient method for assessing the recombination rate of photogenerated electrons and holes by monitoring the decay of photovoltage *V*_*oc*_ over time after illumination termination. The lifetime of the photoelectrons is directly related to the voltage decay, as described by the following equation^40, 41^:

3 $$\tau =\frac{{k}_{B}T}{e}{(\frac{d{V}_{oc}}{dt})}^{-1}$$

where *τ* is the potential-dependent lifetime, *k*_B_ is the Boltzmann constant, *T* is the temperature (K), *e* is the electronic charge, and d*V*_oc_/d*t* is the open-circuit voltage decay over time. Figure 4b shows a comparison of the voltage decay rate of WS_2_ samples without laser treatment and after one, two, or three laser treatments. Laser-treated samples showed lower voltage decay rates, indicating prolonged electron lifetime.

XPS characterization was performed to investigate the chemical composition and band states of the samples. Figure 4c shows the XPS spectrum of WS_2_ film without laser treatment, which contained peaks located at 33.18 and 35.28 eV corresponding to W 4f _7/2_ and W 4f _5/2_ lines of the W atoms in +4 form, respectively^42, 43^. The peak located at 38.38 eV can be ascribed to W 5p _3/2_^44, 45^. The XPS spectrum of a laser-treated sample is shown in Fig. 4d, where peaks located at 32.16 and 34.37 eV belong to W 4f _7/2_ and W 4f _5/2_ peaks of the atoms in +4 form, and relatively high energies of 35.74 and 37.75 eV originate from W 4f _5/2_ and W 4f _7/2_ peaks of the W atoms in +6 oxidation state, all of which are in agreement with previously reported WO_3_ values^20, 46^. These results altogether reveal that the laser-treated sample contained two compositions of chemical compounds (WS_2_ with W^4+^ and WO_3_ with W^6+^). Because the laser-drilling process was conducted in ambient air, partial oxidation of WS_2_ into WO_3_ naturally occurred during localized bond breakage and new bond formation^47^.

Raman images obtained from the samples before (black) and after (red) laser drilling are shown in Fig. 4e. To characterize the impact of laser modification, the Raman spectrum after laser treatment shown in Fig. 4e is from the edge of the laser-drilled hole; the modified sample spectrum (red) showed peaks at 262.5 cm^−1^, 710.2 cm^−1^, and 809.4 cm^−1^. The figure inset shows the magnified spectrum of two peaks: One from 240 to 290 cm^−1^ and the other from 750 to 900 cm^−1^ for a clear comparison. The two peaks between 600 to 900 cm^−1^ can be ascribed to the stretching ν(O-W-O) mode, and the peak at 262.5 cm^−1^ corresponds to the bending δ(O-W-O) mode^48, 49^, indicating partial oxidation of WS_2_ into WO_3_ at the laser-drilled hole edge. These characteristic Raman peaks observed agree well with previous studies of WO_3_^50, 51^. The structural analysis and phase change in the pristine WS_2_ film and laser treated WS_2_ film was examined by X-ray diffractometer (XRD, See Supplementary Fig. S7). Both XRD pattern exhibits the characteristic diffraction peaks corresponding to (002), (004), (101), (103) planes, which match well with standard JCPDS for the 2H-WS_2_ (JCPDS Card No. 08-0237). After laser treatment there are new peaks appear which match the orthorhombic WO_3_ (JCPDS Card No. 32-1394). The low intensity of the WO_3_ peaks, we believe, is due to the small portion of WO_3_ in the sample. Furthermore, it is well known that the WO_3_ can react with KOH and dissolved in KOH aqueous solution^52^, so experiments were conducted to compare the TEM results for investigation of the laser drilled samples influenced by KOH etching (See detailed experiment and TEM results in Fig. S8). The result shows a clearer surface of laser drilled WS_2_ film with cavities after the KOH etching, which also confirms the formation of WO_3_ in WS_2_ film by laser treatment.

The formation of a WS_2_/WO_3_ heterojunction also contributed to the significant enhancement in photocurrent density in the laser-drilled samples. The UV-Vis transmittance spectra of WS_2_ film before and after laser treatment were measured. The band gap energy (E_g_) was evaluated to be 1.4 eV from the plot of (αhν)^1/2^ vs. hν (see Fig. S9 and detailed calculation in Supplementary), which is consisted with previous works^53, 54^. Compared with pristine WS_2_, a slight increase in the band gap of the WS_2_ film after laser treatment indicates the occurrence of oxidation. The approximate valence band position was evaluated based on the flat-band potential from the Mott-Schottky plot (see Fig. S10 and details in Supplementary). The valence band after laser treatment increased, which is probably due to the partial oxidation of WS_2_ into WO_3_ created by laser treatment. WO_3_ has a band gap of 2.5–2.8 eV, of which the potential at the valence band maximum versus the reversible hydrogen electrode (RHE) is larger than that of WS_2_^20, 55^. The approximate band structure and charge transfer of WS_2_/WO_3_ heterostructure is presented in Fig. 4f according to the obtained bandgaps and flat-band potential. The photogenerated electrons were transferred from WS_2_ to WO_3_ through the CB gradient. The valence band (VB) maximum of WS_2_ is lower than that of WO_3_, so holes were able to transfer through the opposite direction and accumulate on the WS_2._ The conveying and separation of visible-light excited charge carriers between different energy levels improved the photocurrent density of laser-drilled samples and slowed the recombination of photogenerated charge pairs; our OCPVD results further support for this observation.

**Figure 5:**
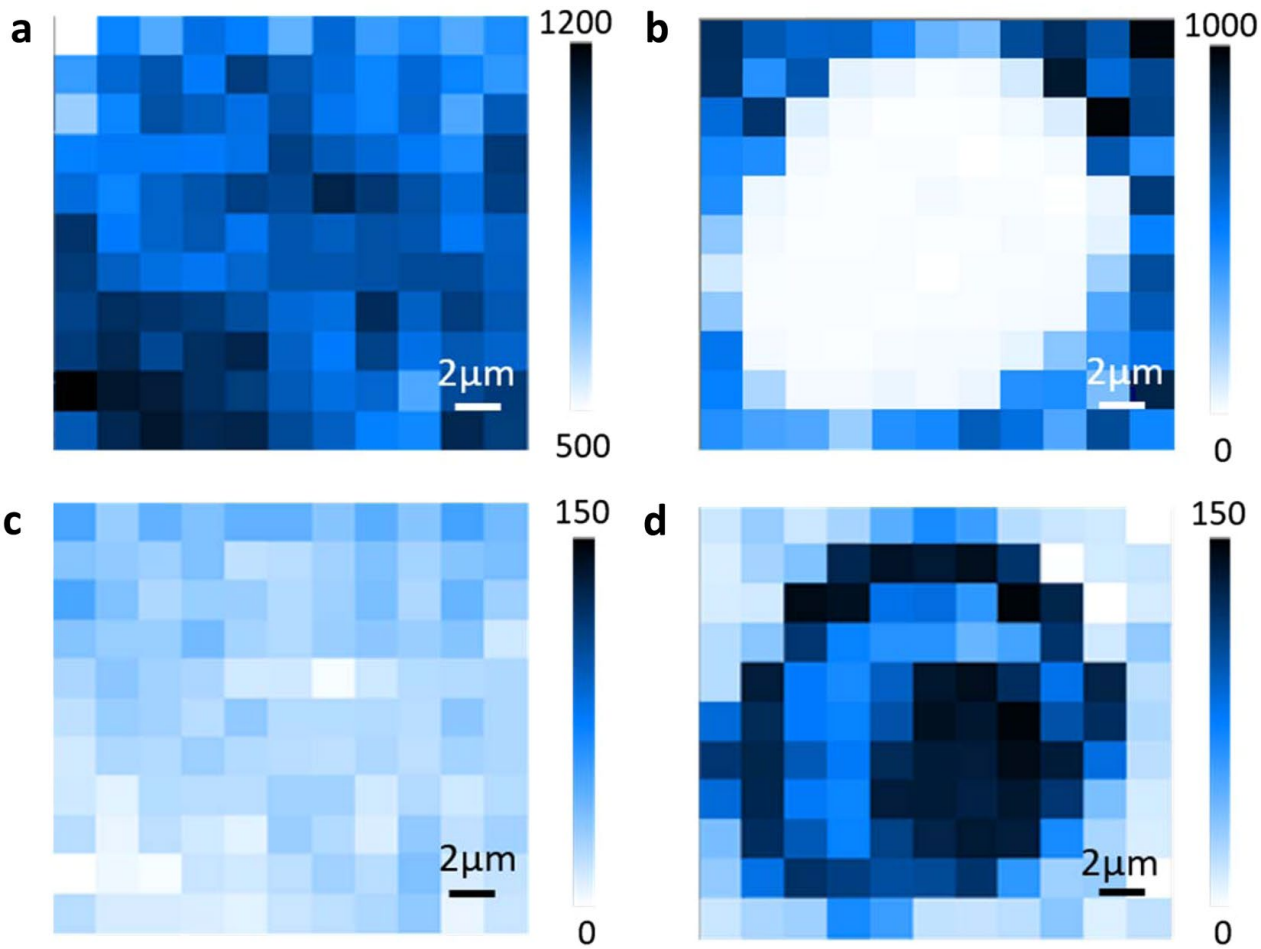
Raman mapping images of WS_2_ film (**a**) before and (**b**) after laser treatment at wavelength of 356 cm^−1^ (representing the existence of WS_2_); (**c**) before and (**d**) after laser treatment at wavelength of 710 cm^−1^ (representing the existence of WO_3_). Darker region means stronger signals.

Raman mapping was further conducted to probe heterojunction locations, which would in practice create a foundation for the controllable and precise positioning of the WS_2_/WO_3_ heterojunction. Figure 5a and b show the intensity distribution of the Raman peak at ~356 cm^−1^ representing the existence of WS_2_ over a selected area of 20 × 20 μm for the samples before and after laser treatment. The shaded area represents the intensity level, where darker shading indicates greater intensity. There was practically no WS_2_ left within the laser-drilled hole area. Figure 5c and d show the corresponding Raman mappings of the typical peak of WO_3_ at ~710 cm^−1^ before and after laser treatment. The darker color distributed around the hole area in Fig. 5d compared to Fig. 5c marks the existence of WO_3_ on the laser treated surface, which further confirms the enhanced partial oxidation induced by laser treatment.

In this study, we utilized laser treatment to create and fine-tune a porous surface structure with increased active edge sites and partial oxidation for the facile formation of WS_2_/WO_3_ heterojunctions. The density of laser-drilled holes increased alongside repeated laser treatments, which enhanced the oxidation and thus the photoelectrocatalytic performance of the sample. After three laser treatments, enhanced photoelectrical activity was observed in regards to the photocurrent density of the WS_2_/WO_3_ heterojunction which improved up to 31 times alongside prolonged electron lifetime. The optimal photocurrent density was ~2 × 10^−5^ Acm^−2^ under visible-light illumination – about 80 times higher than that of pristine sputter-deposited WS_2_ film. This laser-drilling-based heterojunction tailoring process may provide a new and highly effective approach to enhancing the photoelectrocatalytic performance of 2D TMDs materials for electrochemical energy applications.

## Methods

### Preparation of WS_2_ films and laser treatment

WS_2_ thin films were deposited onto a silicon wafer by magnetron sputtering (KYKY, FD-600K) with a 99.99% WS_2_ disk target (Ø*0.125 inch, China New Metal Materials Technology Co, Ltd). The radio frequency power, argon gas pressure, substrate temperature, and deposition time were set as 60 W, 50 Pa, 200 °C, and 10 min, respectively. The sputtered WS_2_ thin films were placed at the center of a tubular furnace with constant temperature of 800 °C to crystallize for 2 h with flowing sulfer/argon ambience in the chamber, then cooled naturally to room temperature. The laser treatment process was conducted with a home-made, nanosecond pulse laser drilling setup equipped with an infrared light laser (power = 0.92 W, λ = 1064 nm, pulse duration = 47 ns, frequency = 50 kHz, laser mode: TEM_00_). A computer was connected to the laser setup to control the laser drill position and area.

### Characterization of laser-dilled porous WS_2_/WO_3_ heterojunction

Optical images were recorded by an optical microscope equipped with a CCD camera (Lecia, DM1750M). The morphology of the WS_2_ film before and after laser treatment was characterized by scanning electron microscope (JEOL, JSM-6490). Field-emission transmission electron microscope (JEOL, JEM-2100F) were used to observe the nanoscale morphology and crystal structure of thin solid film samples before and after laser treatment. The surface topography and roughness of the thin films were examined under an atomic force microscope (Bruker, Nanoscope Multimode 8), and the crystalline compositions and heterojunction locations of the samples were evaluated via Raman spectroscopy (Horiba Jobin Yvon, HR800) with an excitation laser source of 488 nm. X-ray photoelectron spectroscopy (Thermo Scientific, ESCALAB 250Xi) was carried out with a monochromatic Al Kα source to investigate the chemical states of the thin film samples. Mott-Schottcky analysis was conducted using an electrochemical workstation (Zahner, Zennium) with a frequency of 1kHz in dark condition. The structural analysis and phase change of sample was examined by X-ray diffraction (XRD, Rigaku SmartLab) using Cu Kα radiation.

### Photoelectrocatalytic measurements of laser-dilled porous WS_2_/WO_3_ heterojunction

Photoelectrocatalytic measurements were taken in a 5 cm × 5 cm × 5 cm quartz reactor applying the conventional three-electrode system with a Pt-wire as the counter eletrode, Ag/AgCl as the reference electrode, and fabricated samples as the working electrode. A 300 W Xe lamp equipped with a 420 nm cut-off filter (PLS-SXE300, Beijing Perfect Light Technology Co., Ltd.) served as the irradiation source, and the intensity of visible light was measured with an illumination meter (THORLABS, S314C). An electrochemical analyzer (CHI600C, Shanghai Chenhua Instruments Company) was used to record the transient current in 0.1 s intervals as a Xe lamp alternatively switched on/off. Photovoltage decay profiles were measured to determine the recombination rate of photogenerated electrons and holes. Owing to the chemical stability of WS_2_/WO_3_ in acidic electrolyte, 0.5 mol L^−1^ H_2_SO_4_ aqueous solution was used as a supporting electrolyte.

Chemical reagents including sulfer powder (99.5%), potassium hydroxide, sulphuric acid, acetone, and ethanol were purchased from Sigma and used as-received. Throughout the entirety of the experiment, deionized water with a resistivity of 18.2 MΩ cm was used to prepare aqueous solutions and for rinsing.

## Electronic supplementary material

Supplementary Information
