# Supplementary material for: Enhanced Photocatalytic Activity of WS2 Film by Laser Drilling to Produce Porous WS2/WO3 Heterostructure
Source: Sci Rep. 2017 Jun 9;7:3125. doi: 10.1038/s41598-017-03254-2 (PMC5466614; doi:10.1038/s41598-017-03254-2)
Supplement: Supplementary file 1 — Supplementary Information [file 41598_2017_3254_MOESM1_ESM.pdf]

Supporting Information for

Enhanced Photocatalytic Activity of WS_2 Film by Laser Drilling to Produce Porous WS_2/WO_3 Heterostructure

Sainan Ma[1], Longhui Zeng[1,2], Lili Tao[1,2], Chun Yin Tang[1], Huiyu Yuan[1], Hui Long[1], Ping Kwong Cheng[1], Yang Chai[1], Chuansheng Chen[1], Kin Hung Fung[1], Xuming Zhang[1], Shu Ping Lau[1], Yuen Hong Tsang[1,2*]

[1] Department of Applied Physics and Materials Research Center, The Hong Kong Polytechnic University, Hung Hom, Kowloon, Hong Kong, People's Republic of China

[2] The Hong Kong Polytechnic University Shenzhen Research Institute, Shenzhen, People's Republic of China

* E-mail: yuen.tsang@polyu.edu.hk

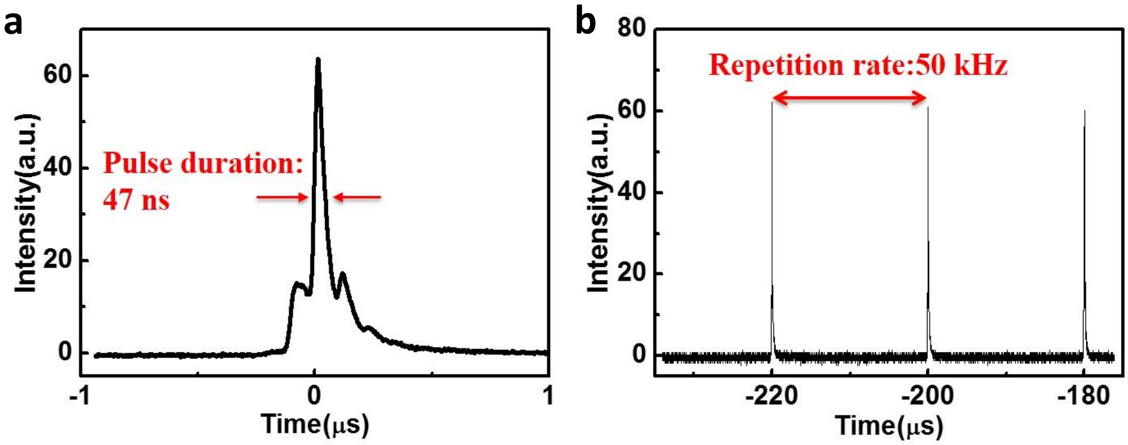

Figure S1. Oscilloscope trace shows (a) the repetition rate of the Q-switched Yb fiber laser and (b) corresponding pulse duration.

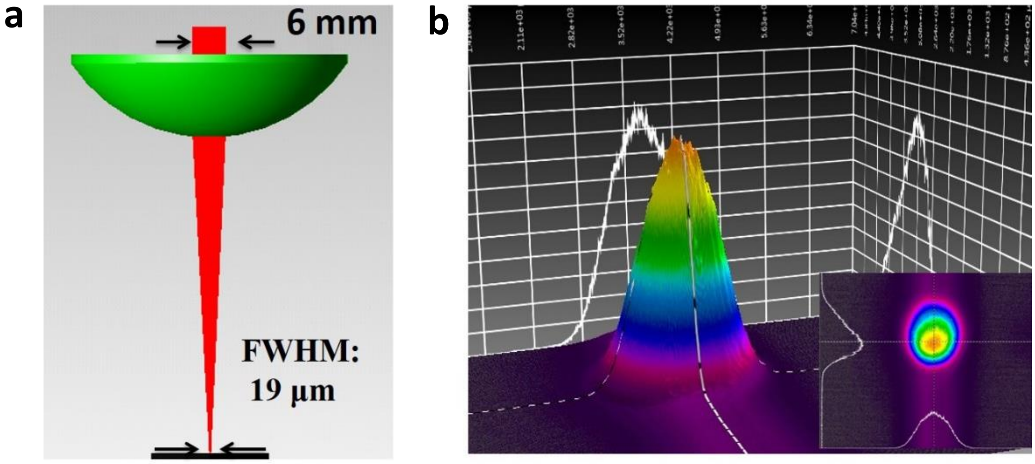

Figure S2. (a) Simplified 3D model of the laser focusing system and (b) the beam profile of the laser spot (near the focus).

Figure S2 shows the laser focusing system and the laser beam profile used for this experiment. The average output power and calculated FWHM laser diameter at focus are about 0.92 W and 19 µm. With 47 ns pulse duration and 50 kHz repetition rate shown in Fig. S1, the calculated pulse energy and peak power are ~18 µJ and ~391 W, respectively. The pulse peak power of the Q-switched nanosecond laser is high enough to produce regular holes on the WS_2 sample. Due to the heat transfer on the sample, the produce hole size is larger than the laser beam diameter.

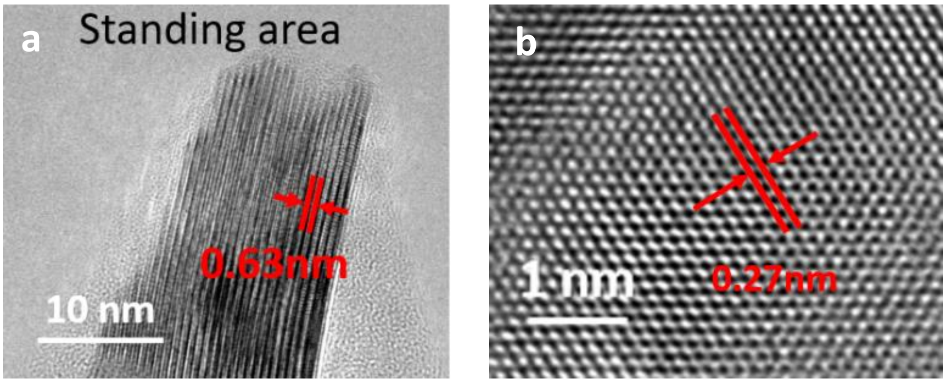

Figure S3. HRTEM images of (a) vertical aligned layers and (b) horizontal aligned layers.

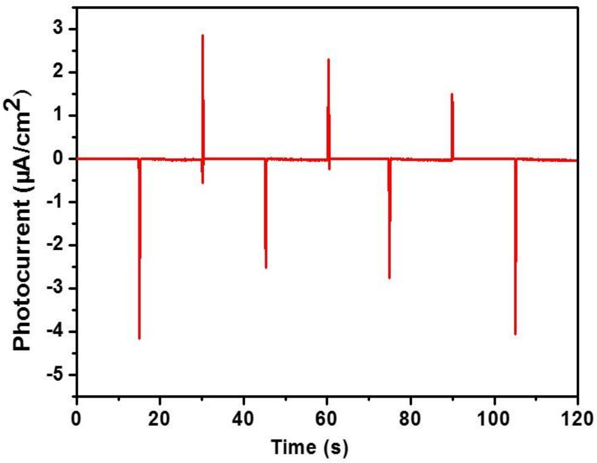

Figure S4. Photocurrent as a function of time under solar simulated illumination (420 nm cut-off) without bias of silicon substrate without laser treatments.

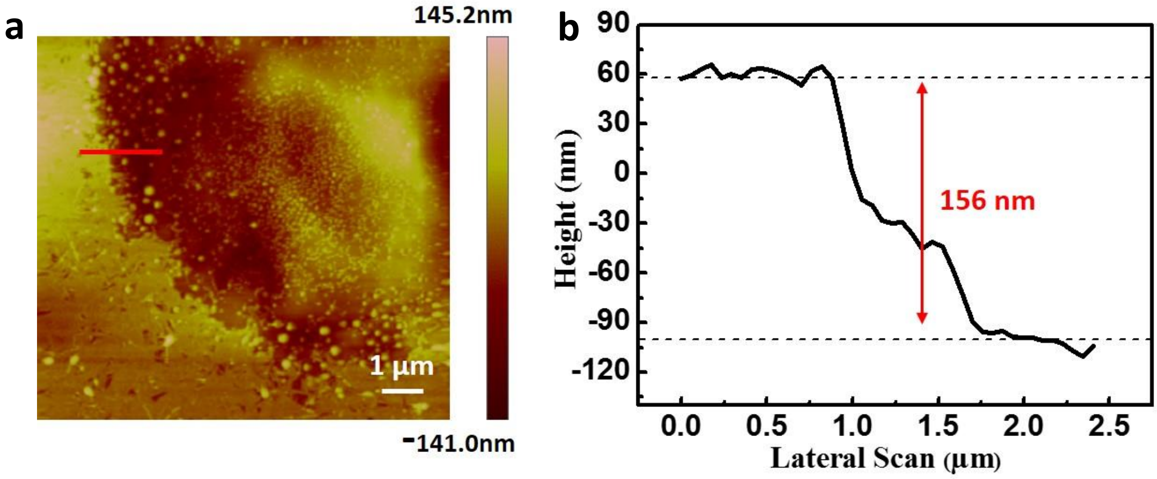

Figure S5. (a) AFM image of hole depth of laser drilled WS_2 film. (b) Height information along the red line marked in (a).

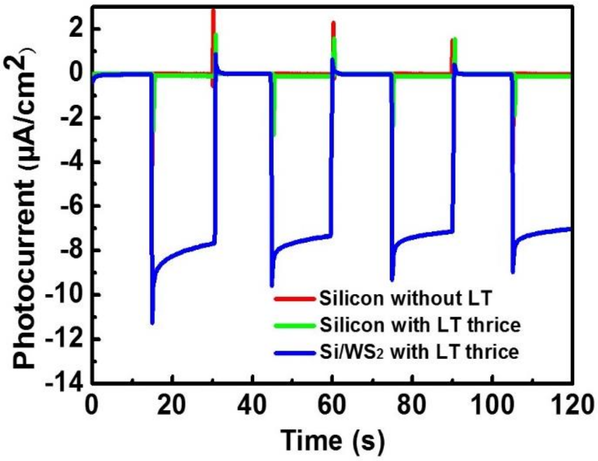

Figure S6. Photocurrent as a function of time under solar simulated illumination (420 nm cut-off) without bias of the corresponding pure silicon substrate without laser treatment (red curve), pure silicon substrate with laser treatment three times (green curve) and Si/WS_2 sample with laser treatment three times (blue curve). (LT represents laser treatment)

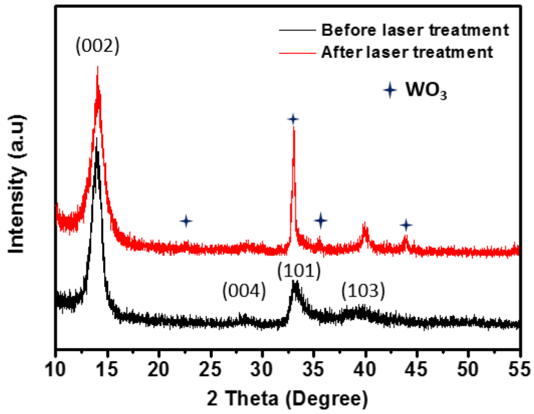

Figure S7. XRD pattern of WS_2 film before laser treatment (black curve) and after laser treatment 20 times (red curve).

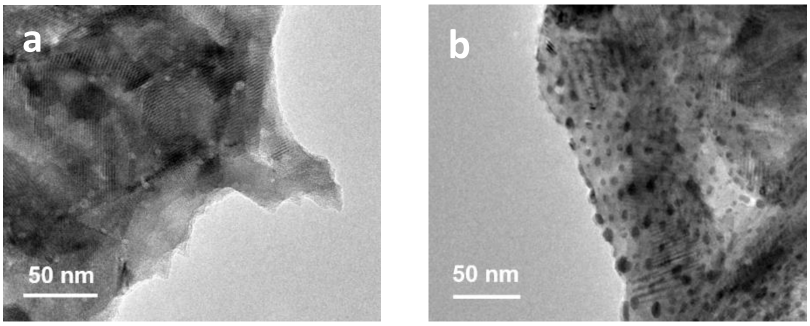

Figure S8. TEM images of laser-drilled WS_2 film a) with and b) without KOH etching.

The TEM sample in Fig. S8a was prepared by immersing the laser drilled WS_2 (LD-WS_2) film in potassium hydroxide (KOH) after spin-coating a layer of PMMA. KOH can etch the Si substrate and the PMMA/LD-WS_2 film will float in the solution. After being washed with DI water for three times, the floating PMMA/LD-WS_2 film was transferred to the copper grid for TEM observation. For the sample preparation in Fig. S8b, the WS_2 sample was transferred to copper grid and then the laser drilling was carried out directly on the copper grid. Afterwards, the sample was directly used for TEM observation. As shown in Fig. S8b, there are numerous nanoparticles on the WS_2 film around the laser drilled hole, which can not be observed in Fig. S8a. Furthermore, some relatively light points can be observed in the Fig. S8a. As KOH can react with WO_3 to form the K_2WO_4 which is dissolvable in aqueous solution, the difference in Fig. S7a and b suggests that the dark particles are the WO_3 created by laser. These WO_3 can dissolved into the KOH solution resulting in a clearer film surface with the light dots. This result indicates that the nanoparticles around the hole edge should be WO_3.

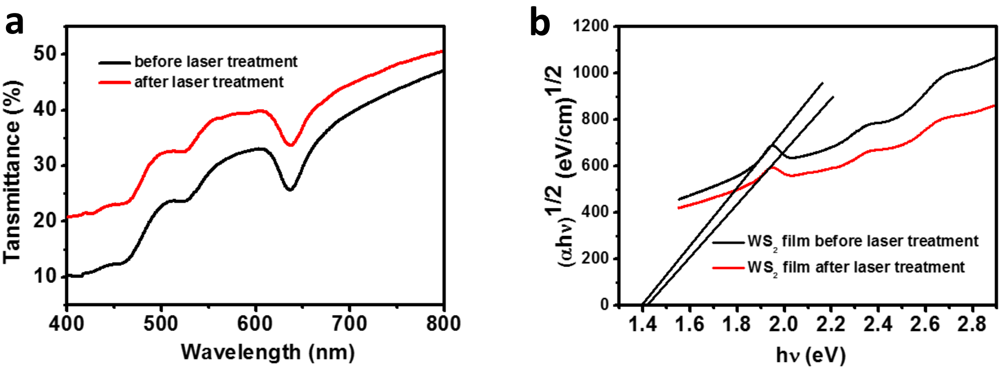

Figure S9. a) UV-Vis transmittance spectrum of WS_2 film before and after laser treatment. b) A plot transformed according to the Tauc's relation versus energy of light.

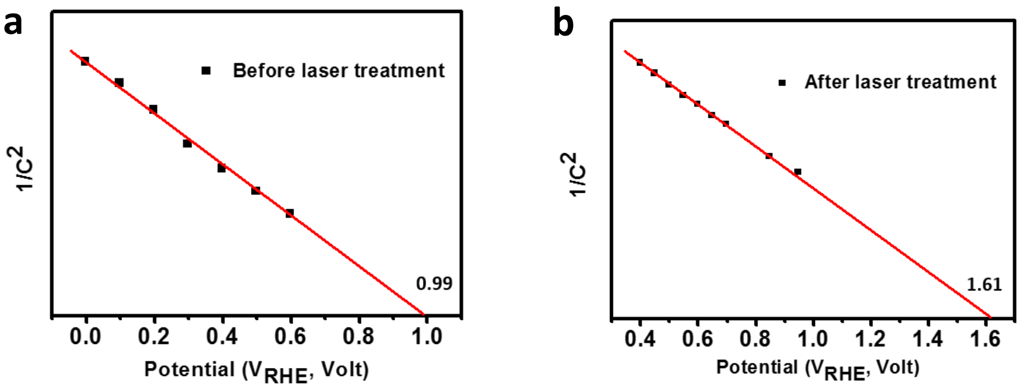

Figure S10. Mott-Schottky plots of WS_2 film a) before laser treatment and b) after laser treatment.

The band gap energy (E_g) was evaluated by using the Tauc's relation: $(\alpha h\nu)^{1/n} = A\ (h\nu - E_g)$ where hν is the incident photo energy, n= 2 or ½ depending upon whether the transition is indirect or direct, respectively and α is the absorption coefficient.[1,2] The absorption coefficient α is calculated using the data from the UV-Vis transmittance: $\alpha = 1/d \ln (1/T)$, where d is the film thickness and T is the transmittance.[2] In order to measure the UV-Vis transmittance spectra, the WS_2 film was deposited by sputtering on quartz and annealed under the same process. Here, WS_2 in multiple layer is considered as indirect semiconductors,[3,4] we can calculate the band gap E_g from the plot $(\alpha h\nu)^{1/2}$ vs. hν. The value was measured to be 1.4 eV.

Figure S10 shows the Mott-Schottky plots of the WS_2 film before and after laser treatment. $1/C^2$ is the function of electrochemical potentials, in which the capacitance C is obtained by measuring the impedance. The flat-band potentials can be estimated from the intercepts of the extrapolated lines in the Mott-Schottky plots. The negative slopes of Mott-Schottky plots indicate that the WS_2 is p-type. For p-type semiconductors, the Fermi level is located close to the valence band edge, so the flat-band potential is assumed to be closed to the valence band edge.[5,6] The approximate band-edge position was calculated based on the measured bandgap and flat-band potential.

Reference

1 Jana, S., Bera, P., Chakraborty, B., Mitra, B. C. & Mondal, A. Impact of annealing on the electrodeposited WS_2 thin films: Enhanced photodegradation of coupled semiconductor. *Appl. Surf. Sci.* **317**, 154-159 (2014).

2 Adelifard, M., Salamatizadeh, R. & Ketabi, S. Fabrication and characterization of nanostructural WS_2/WO_3 binary compound semiconductors prepared by the sulfurization of sprayed thin films. *J. Mater. Sci. Mater. Electron.* **27**, 5243-5250 (2016).

3 Mak, K. F., Lee, C., Hone, J., Shan, J. & Heinz, T. F. Atomically thin MoS_2: a new direct-gap semiconductor. *Phys. Rev. Lett.* **105**, 136805 (2010).

4 Zhao, W. *et al.* Origin of indirect optical transitions in few-layer MoS_2, WS_2, and WSe_2. *Nano Lett.* **13**, 5627-5634 (2013).

5 Kocha, S. S., Turner, J. A. & Nozik, A. Study of the Schottky barrier and determination of the energetic positions of band edges at the n-and p-type gallium indium phosphide electrode| electrolyte interface. *J. Electroanal. Chem.* **367**, 27-30 (1994).

6 Liao, L. *et al.* Efficient solar water-splitting using a nanocrystalline CoO photocatalyst. *Nat Nanotechnol.* **9**, 69-73 (2014).
